# The Provision of Dental Care to COVID-19 Survivors: A Concise Review

**DOI:** 10.1016/j.identj.2022.05.009

**Published:** 2022-06-01

**Authors:** Wei Cheong Ngeow, Liszen Tang, Jan Yang Ho, Hui Wen Tay, Raymond Chung Wen Wong, Mas Suryalis Ahmad, Vinay Marla, Karthick Sekar

**Affiliations:** a Faculty of Dentistry, University of Malaya, Kuala Lumpur, Malaysia; b Oral & Maxillofacial Surgery Unit, School of Dental Sciences, Universiti Sains Malaysia, Kelantan, Malaysia; c Department of Restorative Dentistry, School of Dentistry, International Medical University, Kuala Lumpur, Malaysia; d Department of Oral & Maxillofacial Surgery, Faculty of Dentistry, National University of Singapore, Singapore; e Faculty of Dentistry, Universiti Teknologi MARA, Sungai Buloh Campus, Selangor Darul Ehsan, Malaysia; f Faculty of Dentistry, Lincoln University, Selangor Darul Ehsan, Malaysia

**Keywords:** COVID-19, Dental management, Pulmonary, Cardiovascular, Long COVID

## Abstract

**Aims:**

It has been reported that there are a certain percentage of COVID-19 patients who recover but suffer from devastating permanent organ damage or failure. Others suffer from long Covid syndrome, with prolonged symptoms that persist more than 12 weeks. However, there is scarcity of literature regarding the provision of dental treatment for these two groups of patients. This manuscript reviews the impact of multi-system involvement on the provision of dental care to these patients.

**Materials and methods:**

A search of literature was done in PubMed-Medline and Scopus databases to review the available literature on COVID-19 impacts on pulmonary, cardiovascular, haematologic, renal, gastrointestinal, endocrine, and neurologic systems and respective management in dental clinical settings.

**Results:**

The literature search from PubMed-Medline and Scopus databases resulted in 74 salient articles that contributed to the concise review on COVID-19 effects on pulmonary, cardiovascular, haematologic, renal, gastrointestinal, endocrine, and neurologic systems and/or its respective dental management recommendations.

**Conclusions:**

This concise review covers the management of post COVID-19 patients with pulmonary, cardiovascular, haematologic, renal, gastrointestinal, endocrine, or neurologic system complications.

## Introduction

**Fig. 1:**
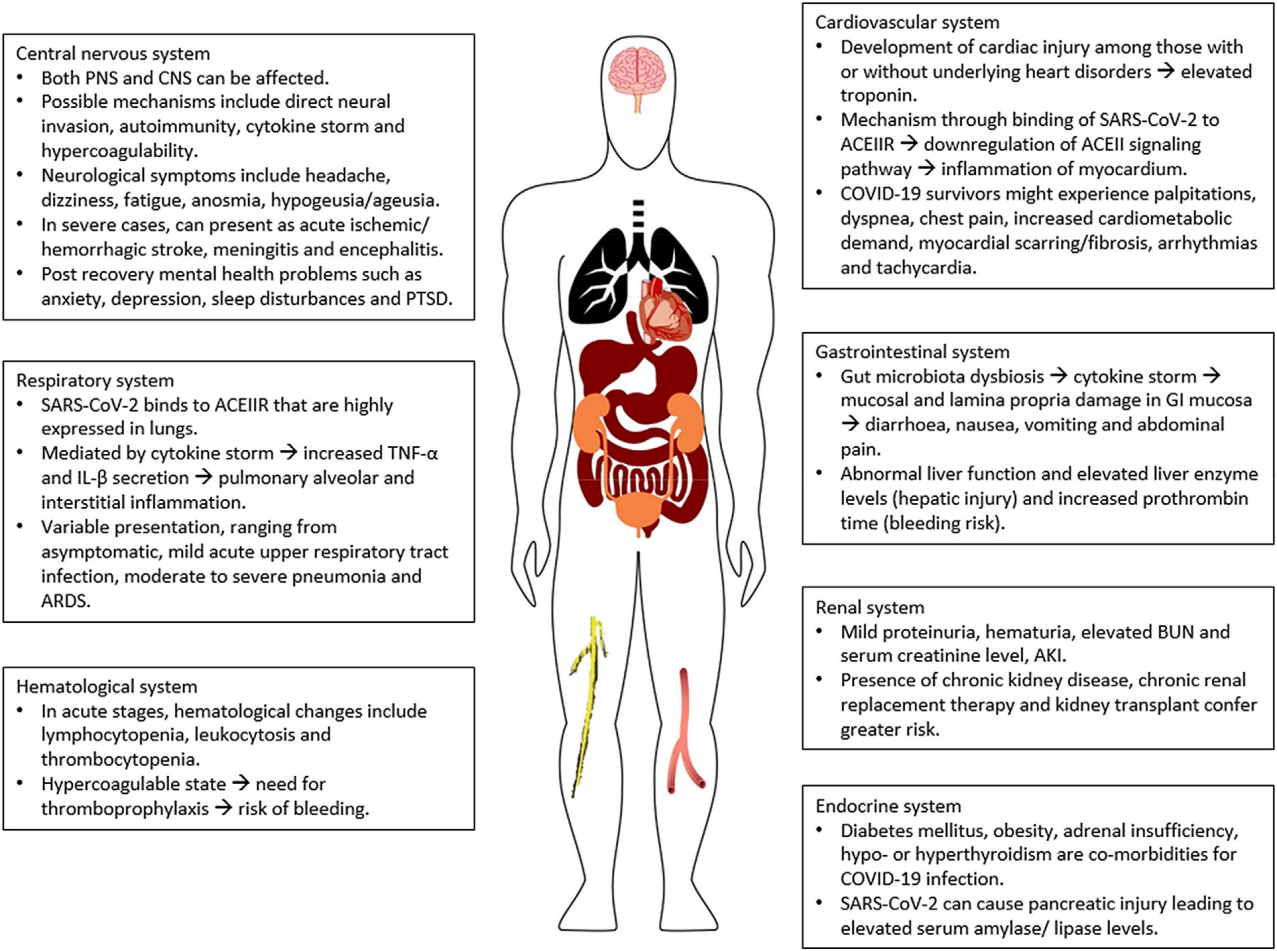
The effect of COVID-19 infection on various body systems.

ACEIIR, angiotensin-converting enzyme inhibitor II receptor; AKI, acute kidney injury; ARDS, acute respiratory distress syndrome; BUN, blood urea nitrogen; CNS, central nervous system; GFR, glomerular filtration rate; GI, gastrointestinal; PNS, peripheral nervous system; PTSD, posttraumatic stress disorder; SARS-CoV-2, severe acute respiratory syndrome coronavirus 2.

Severe acute respiratory syndrome coronavirus 2 (SARS-CoV-2) is transmitted via air droplets, with clinical manifestations that range from asymptomatic to multiorgan system dysfunction.^1^ The multisystemic nature of the disease is related to the tropism of the virus for the angiotensin-converting enzyme 2 (ACE-2) receptors in several organs.^2^ The highest levels of SARS-CoV-2 copies are detected in the respiratory tract, with lower levels detected in the kidney, liver, heart, brain, and blood. Multisystem involvement in COVID-19 infection can range from mild to severe devastating permanent organ damage or failure (Figure 1).^2^ Patients are defined as postacute COVID-19 patients if they present with persistent symptoms and/or long-term complications of SARS-CoV-2 infection beyond 4 weeks from the onset of symptoms. They can be further divided into subacute or ongoing symptomatic (4–12 weeks) and chronic or post-COVID-19 syndrome (>12 weeks).

Current management against COVID-19 includes vaccination and/or treatment of systemic symptoms. Alharbi et al in their proposed guideline of dental care provision stated that convalescent recovered patients should be considered as potential SARS-CoV-2 carriers for at least 30 days after laboratory test confirmation of recovery.^3^ Some of them have been reported to experience persistent immune suppression and altered cholesterol metabolism, blood coagulation, and cardiomyopathy, which appear to be a prelude to long COVID.^4^

COVID-19 survivors have been reported to manifest up to 200 symptoms. A cohort study found that oral manifestations after recovery are not uncommon.^5^ They include salivary gland ectasia (38%), xerostomia (30%), masticatory muscle weakness (19%), loss of taste and smell (10%), and temporomandibular joint abnormalities (7%).^6^

Currently, one report highlighting the management of dental care in COVID-19 survivors suggested monitoring oxygen saturation, heart rate, and blood pressure as part of their routine examination during dental treatment.^7^ A physical performance test and psychologic screening may be performed when applicable.^7^ Chakraborty et al suggested that future research should be done on the effects of thromboprophylaxis in dental treatment and the safe administration of perioperative antibiotics and analgesics in COVID-19 survivors.^7^ The current concise review tries to address these issues as well as other medical complications during the dental treatment of these patients.

## Methods

A literature search was done in PubMed-MEDLINE and Scopus databases using the keywords “SARS-CoV-2,” “COVID-19,” “severe acute respiratory syndrome,” “dental management,” “pulmonary,” “cardiovascular,” “neurological,” “renal,” “gastrointestinal,” “endocrine,” “haematological,” “COVID-19 vaccine,” “COVID-19 vaccine booster,” “unvaccinated,” and “anti-vaccinated” to identify the literature published in English between January 2000 and March 2022 (Table 1). This concise review aims to identify the relevant dental management of post-COVID-19 patients or patients with long COVID syndrome from currently available published studies. The inclusion criteria are articles of clinical studies such as randomised control studies, cohort studies, and multicentre studies as well as systematic review and meta-analysis due to the high level of evidence. Relevant systemic guidelines and position papers were also included, and the recommendations are extrapolated to dental care where applicable. All the obtained abstracts of titles were screened independently to remove irrelevant articles by 7 authors. The exclusion criteria are those articles of opinion, perspective, commentary, editorial, communication, case report, case series, non–English-language, no full text, and of irrelevant information. Then the collected article abstracts and full texts were critically reviewed. The discrepancies amongst 7 authors would be resolved by iteration. An independent reviewer (first author) who was blinded to other reviewers’ assessment resolved unreconciled discrepancies. The risk of bias assessment was done on all the included articles using methodological quality assessment by combining the proposed criteria of the Preferred Reporting Items for Systematic Reviews and Meta-Analyses (PRISMA) statement,^8^ Risk of Bias in Systematic Reviews (ROBIS),^9^ and the Strengthening the Reporting of Observational Studies in Epidemiology (STROBE) statement.^10^

**Table 1:** Example of the electronic search strategy in PubMed-MEDLINE: advanced search for central nervous system impacts by COVID-19.

Search Strategy	Results
[(COVID-19) OR (SARS-CoV-2)] AND [(central nervous system) OR (peripheral nervous system)] AND [(neurological manifestations) OR (neurological complications)] AND (covid-19 syndrome)	1479 articles

## Results

**Fig. 2:**
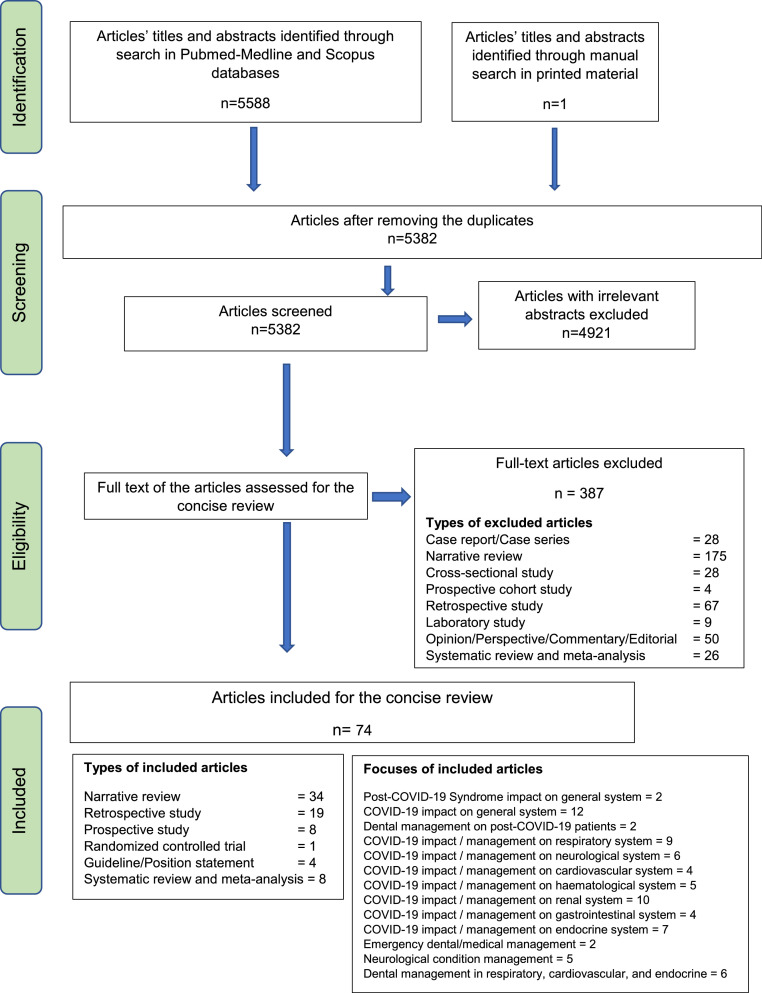
Flowchart of search strategy outcomes.

A total of 5588 studies were retrieved from the PudMed-MEDLINE and Scopus databases, and 1 was from a manual search in printed material. After undergoing screening and eligibility phases, a total of 74 articles were included in the review; 387 articles were excluded at the full-text stage, with classification of the types and focuses of studies as illustrated in Figure 2. The different risk of bias level of all included articles are illustrated in Table 2 and Table 3.

**Table 2:** Risk of bias assessment of the included studies.

Article	Year	Study type	Theme/focus of the paper	Random selection in population	Defined inclusion/exclusion criteria	Loss to follow-up reported	Validated measurement	Statistical analysis	Estimated potential risk of bias
**Nalbandian A et al****^6^**	2021	Narrative review	General systemic impact of post COVID-19 syndrome	No	No	No	No	No	High
**Chakraborty T et al****^7^**	2021	Narrative review	General systemic dental management on post-COVID-19	No	Yes	No	Yes	No	High
**Zhao Y-M et al****^17^**	2020	Retrospective multicentre cohort study	Post-COVID-19 respiratory system	No	Yes	Yes	Yes	Yes	Moderate
**Sonnweber T et al****^18^**	2021	Prospective cohort study	Post-COVID-19 respiratory system	No	Yes	Yes	Yes	Yes	Moderate
**Blomberg B et al****^19^**	2021	Prospective cohort study	Post-COVID-19 respiratory system	No	Yes	Yes	Yes	Yes	Moderate
**Yang L-L et al****^20^**	2020	Narrative review	Post-COVID-19 respiratory rehabilitation	No	No	No	No	No	High
**Carvalho AC et al****^21^**	2021	Randomised controlled trial	Post-COVID-19 respiratory rehabilitation	Yes	Yes	Yes	Yes	Yes	Low
**Jevon P^22^**	2014	Narrative review	Emergency in dentistry	No	No	No	No	No	High
**Devlin J^23^**	2014	Narrative review	Dental management for respiratory system	No	No	No	No	No	High
**O'Driscoll BR et al****^24^**	2017	Guideline	Respiratory emergency management	No	Yes	No	Yes	Yes	High
**Bolaki M et al****^25^**	2020	Narrative review	COVID-19 respiratory management	No	No	No	No	No	High
**Claramunt Lozano A et al****^26^**	2011	Narrative review	Dental management for respiratory system	No	Yes	No	Yes	No	High
**Leung JM et al****^28^**	2020	Narrative review	COVID-19 and respiratory system	No	Yes	No	Yes	No	High
**Lee SC et al****^29^**	2021	Retrospective cohort study	COVID-19 and respiratory system	No	Yes	Yes	Yes	Yes	moderate
**Huang C et al****^31^**	2020	Prospective cohort study	COVID-19 impact on the general system	No	Yes	No	Yes	Yes	Moderate
**Chen N et al****^32^**	2020	Retrospective descriptive study	COVID-19 impact on the general system	No	Yes	No	Yes	Yes	Moderate
**Klopfenstein T et al****^33^**	2020	Retrospective observational study	COVID-19 impact on the neurologic system	No	Yes	No	Yes	Yes	Moderate
**Chou SH-Y et al****^34^**	2021	Multicohort observational study	COVID-19 impact on the neurologic system	No	Yes	No	Yes	Yes	Moderate
**Gupta A et al****^35^**	2020	Review article	COVID-19 impact on the general system	No	No	No	No	No	High
**Tang N et al****^37^**	2020	Retrospective cohort study	COVID-19–related coagulopathy and its management	No	No	Yes	Yes	Yes	High
**Paranjpe I et al****^38^**	2020	Retrospective Cohort study	COVID-19–related coagulopathy and its management	No	No	No	Yes	Yes	High
**McCaul JA et al****^40^**	2014	Review article	Bell's palsy management (non-COVID-19–related)	No	No	No	No	No	High
**Baugh RFet al****^41^**	2013	Clinical practice guideline	Bell's palsy clinical practice guideline	No	No	No	No	No	High
**Thieben MJ et al****^42^**	2007	Retrospective cross-sectional study	Postural orthostatic tachycardia syndrome and its management (non-COVID-19–related)	No	Yes	No	Yes	Yes	High
**Raj SR et al****^43^**	2020	Position statement	Position statement on postural orthostatic tachycardia syndrome and its management (non-COVID-19–related)	No	No	No	No	No	High
**Moldofsky H et al****^44^**	2020	Retrospective case-control study	Post-COVID-19 infection impact on mental health/psychology	No	No	Yes	Yes	Yes	High
**Madjid M et al****^45^**	2020	Narrative review	COVID-19 impact on cardiovascular system	No	No	No	No	No	High
**Polito MV et al****^46^**	2021	Narrative review	Sequelae of COVID-19 on cardiovascular system	No	No	No	No	No	High
**Visco V et al****^47^**	2022	Narrative review	COVID-19 impact on multiple systems	No	No	No	No	No	High
**Caspersen IH et al****^48^**	2022	Narrative review	symptoms after COVID-19	No	No	No	No	No	High
**Raman B et al****^49^**	2022	Narrative review	Sequelae of COVID-19 on cardiovascular system	No	No	No	No	No	High
**Wu L et al****^50^**	2022	Narrative review	Symptoms after COVID-19 amongst hospitalised patients	No	No	No	No	No	High
**Akbari A et al****^51^**	2022	Narrative review	Symptoms requiring readmission post-COVID-19	No	No	No	No	No	High
**Joshee S et al****^54^**	2022	Narrative review	Long-term effects of COVID-19	No	No	No	No	No	High
**Sanyaolu A et al****^55^**	2022	Narrative review	Sequelae of COVID-19 on multiple systems	No	No	No	No	No	High
**Elseidy SA et al****^56^**	2022	Narrative review	Sequelae of COVID-19 on cardiovascular system	No	No	No	No	No	High
**Wright C^57^**	2019	Clinical practice guideline	Practice guideline on patient management	No	No	No	No	No	High
**Cheung CKM et al****^58^**	2021	Narrative review	Haematology and COVID-19	No	No	No	No	No	High
**García de Guadiana-Romualdo L et al****^59^**	2021	Prospective observational study	Haematology and COVID-19	No	Yes	Yes	Yes	Yes	Moderate
**Letícia de Oliveira Toledo S et al****^60^**	2020	Narrative review	Haematology and COVID-19	No	No	No	No	No	High
**Terpos E et al****^61^**	2020	Narrative review	Haematology and COVID-19	No	No	No	No	No	High
**Korompoki E et al****^62^**	2022	Narrative review	Haematology and COVID-19	No	No	No	No	No	High
**Bowe B et al****^64^**	2021	Prospective cohort study	COVID-19 impact on the renal system	No	No	No	Yes	Yes	High
**Abdallah E et al****^65^**	2021	Prospective observational study	COVID-19 impact on the renal system	No	No	No	No	Yes	High
**Arikan H et al****^66^**	2021	Retrospective observational study	COVID-19 impact on the renal system	No	Yes	No	Yes	Yes	High
**Bowe B et al****^67^**	2020	Prospective cohort study	COVID-19 impact on the renal system	No	Yes	Yes	Yes	Yes	Moderate
**Chan L et al****^68^**	2020	Retrospective observational study	COVID-19 impact on the renal system	No	Yes	No	Yes	Yes	High
**Almeida DC De et al****^69^**	2021	Retrospective cohort study	COVID-19 impact on the renal system	No	Yes	No	Yes	Yes	High
**Yu Y et al****^70^**	2021	Retrospective cohort study	COVID-19 impact on the renal system	No	No	No	Yes	Yes	High
**D'Amico F et al****^82^**	2020	Narrative review	COVID-19 and gastrointestinal system	No	No	No	No	No	High
**Zhong P et al****^83^**	2021	Narrative review	COVID-19 and gastrointestinal system	N/A	No	No	No	No	High
**Marazuela M et al****^84^**	2020	Narrative review	COVID-19 and endocrine system	No	No	No	No	No	High
**Scappaticcio Let al****^85^**	2020	Narrative review	COVID-19 and endocrine system	No	No	No	No	No	High
**Guan WJ et al****^86^**	2020	Retrospective observational study	COVID-19 and general systemic manifestations	No	Yes	No	Yes	Yes	High
**Richardson S et al****^87^**	2020	Retrospective observational study	COVID-19 and general systemic manifestations	No	Yes	No	Yes	Yes	High
**Petrilli CM et al****^88^**	2020	Retrospective observational study	COVID-19 and general systemic manifestations	No	Yes	No	Yes	Yes	High
**Hernández-Galdamez DR et al****^89^**	2020	Retrospective observational study	COVID-19 and general systemic manifestations	No	Yes	No	Yes	Yes	High
**Muniyappa R et al****^90^**	2020	Narrative review	COVID-19 and endocrine system	No	No	No	No	No	High
**Pal R & Bannerjee M.****^91^**	2020	Narrative review	COVID-19 and endocrine system	No	No	No	No	No	High
**Liu F et al****^92^**	2020	Retrospective observational study	COVID-19 and endocrine system	No	Yes	No	Yes	Yes	High
**Chatterjee S et al****^93^**	2020	Narrative review	COVID-19 and endocrine system	No	No	No	No	No	High
**Bhandari S et al****^94^**	2020	Retrospective observational study	COVID-19 and endocrine system	No	Yes	No	Yes	Yes	High
**Pfützner A et al****^95^**	2020	Narrative review	COVID-19 and endocrine system with dental manifestations	No	No	No	No	No	High
**Miller A & Ouanounou A.****^96^**	2020	Narrative review	Dental management associated with endocrine disorders	No	No	No	No	No	High
**Vernillo AT****^97^**	2003	Narrative review	Dental management associated with endocrine disorders	No	No	No	No	No	High
**Yang LC et al****^98^**	2020	Prospective observational study	Dental management associated with respiratory disorders	Yes	Yes	No	Yes	Yes	Moderate

**Table 3:** Risk of bias assessment of the included systematic review and meta-analysis.

Author	Study type	Theme/focus of the article	Study eligibility criteria	Identification and selection of studies	Data collection and study appraisal	Synthesis and findings	Potential risk of bias
*Gerayeli FV et al**^30^* *2021*	Systematic review and meta-analysis	COVID-19 and respiratory system	Yes	Yes	Yes	Yes	Low
*Tan YK et al**^36^* *2020*	Systematic review and meta-analysis	COVID-19 and acute ischemic stroke	Yes	Yes	Yes	Yes	Low
*Gupta S et al**^39^* *2021*	Systematic review	Bell's palsy as the only neurologic presentation of COVID-19	Yes	Yes	Yes	Yes	Low
*Bajwa H et al**^72^* *2020*	Systematic review	Renal involvement in COVID-19	No	No	No	No	High
*Fu EL et al**^63^* *2020*	Systematic review and meta-analysis	Renal involvement in COVID-19	No	No	No	Yes	High
*Kunutsor SK et al**^71^* *2020*	Systematic review and meta-analysis	Renal involvement in COVID-19	Yes	No	No	Yes	High
*Cheung KS et al**^80^* *2020*	Cohort Study and systematic review/metanalysis	COVID-19 and gastrointestinal system	Yes	Yes	Yes	Yes	Low
*Wang J gan et al**^81^* *2020*	Systematic review/metanalysis	COVID-19 and gastrointestinal system	Yes	Yes	Yes	Yes	Low

## Discussion

There is still a lacunae in terms of managing post-COVID-19 dental patients. Balaji highlighted that many of these patients are treated with long-term medications that interact with dentistry-related medications.^11^ He emphasised the need to include history of COVID-19 infection and its residual impact, as this may reveal hidden health issues that may complicate dental care. Chakraborty et al also highlighted the possible challenges that dentists might face and came up with several guidelines.^7^ Nevertheless, there is a dire need for information for the dental practice going forwards. This review attempts to provide some practical suggestions based on available literature and the authors’ experience treating medically compromised patients. The knowledge of post-COVID syndrome makes it important that a basic general evaluation be performed before every procedure and a physician's opinion sought before performing dental work.

### Effect on the respiratory system and how it impacts dental management

Frequent employment of aerosol-generating-procedures in dentistry warrants the need for rigorous infection control measures, even with vaccinated patients.^12^, ^13^, ^14^, ^15^, ^16^ Some patients still present with persistent lung function impairment during convalescence after discharge.^17^, ^18^, ^19^ Therefore, the patient's attending medical physician should be consulted on the ongoing pulmonary rehabilitation programme (that might commence 3 to 6 times per week) and medications taken.^20^^,^^21^ Their elective dental appointment should be arranged on a day without pulmonary rehabilitation to prevent physical overexertion.

The monitoring recommended by Chakraborty et al shall be adopted, as these patients may be susceptible to hypoxemia during a stressful and long dental treatment.^7^ If peripheral capillary oxygen were to drop to less than 88% or they experience symptoms such as profuse sweating, shortness of breath, palpitations, or chest tightness, the dentist should terminate dental treatment immediately and institute high-flow (15 L/min) supplementary oxygen therapy and other medical treatment as indicated, with the aim to restore oxygen saturation to the target range of 88% to 92%.^22^, ^23^, ^24^ Apart from this, COVID-19 survivors prescribed corticosteroids should take their corticosteroids before dental treatment. A supplementary dose of corticosteroids is given to prevent adrenocortical-related emergency.^25^^,^^26^ Patients should be seated upright during dental treatment to reduce the risk of dyspnoeic attack. For patients who are asthmatic, the dental practitioner should avoid using local anaesthetic with adrenaline, as these solutions contain a sulphite preservative that could trigger an asthmatic attack.

In terms of medication prescription, those who are taking theophylline should not be prescribed macrolide antibiotics to prevent symptoms such as nausea, vomiting, or palpitations due to increased serum methylxanthine level.^27^ Narcotic, antihistamine, anticholinergic, and hypnotic agents should not be prescribed, as they might exacerbate chronic obstructive pulmonary disease after COVID-19 infection.^28^, ^29^, ^30^ It is also best to avoid prescribing nonsteroidal anti-inflammatory drugs (NSAIDs) to the recovered COVID-19 patient with asthma to prevent severe bronchospasm.

### Effect of COVID-19 on the central nervous system and how it impacts dental management

There is growing evidence on neurologic complications, which occur more commonly in elderly individuals or people with comorbidities.^6^ Nonspecific mild neurologic symptoms include headache (8%–42%), dizziness (12%), anorexia (40%), myalgia and/or fatigue (11%–44%), anosmia (5%), and hypogeusia or ageusia (5%).^31^, ^32^, ^33^, ^34^ The most prevalent neurologic signs were acute encephalopathy (49%) presenting as altered mental status, abnormal speech (5%) or motor movement (3%), impaired consciousness or coma (17%), and stroke (6%)*.*^34^

Treatment for COVID-19 patients with stroke include the administration of anticoagulants such as low-molecular-weight heparin or unfractionated heparin*.*^35^^,^^36^ For patients who survived the infection, oral anticoagulants are sometimes continued due to prothrombotic state*.*^37^^,^^38^ Routine or elective dental procedures are best deferred to 6 months after the incident of cerebrovascular accident. Special precautions with local haemostatic measures including the placement of local haemostatic agents, suturing, gauze compression, and a prescription of mouthwash 5% tranexamic acid should be undertaken especially for invasive dental procedures. NSAIDS should be avoided in pain management to reduce the risk of bleeding.

Bell's palsy is increasingly being reported as a possible neurologic complication of COVID-19 and its vaccination.^39^ Patients with Bell's palsy are usually started on a 10-day course of corticosteroids.^40^^,^^41^ They would not require corticosteroid supplement before dental procedures due to the short course of corticosteroid therapy.

Some patients might present with persistent autonomic nervous system dysfunction, known as postural orthostatic tachycardia syndrome (POTS). They may complain of palpitations and dizziness, especially upon abrupt standing from a dental chair, or fatigue or blurred vision.^42^ Neurocardiogenic syncope is a situation in which patients experienced loss of consciousness with abrupt blood pressure and heart rate reductions during standing.^43^ Dentists should be fully equipped with the basic algorithm of: position (P), airway (A), breathing (B), circulation (C), and definitive treatment/differential diagnosis/drugs/defibrillation (D), in managing such emergency. As patients with POTS may also be prescribed a low-dose beta blocker, dentists have to look out for gingival hyperplasia, which may follow.^6^

Last, patients’ mental health may be affected after recovery from COVID-19.^7^ Some patients (30%–80%) reported impaired sustained attention, concentration, memory, comprehension, or mental processing speed.^44^ Therefore, they may neglect good oral hygiene or lack the ability to follow instructions following dental treatment. Hence, the dentists and patients’ caregivers should diligently provide oral hygiene maintenance.

### Effect of COVID-19 on the cardiovascular system and how it impacts dental management

Cardiac injury may develop in COVID-19 patients with or without an existing cardiovascular disease.^45^ They need close monitoring, even after discharge, as the ongoing systemic inflammation may lead to ventricular dysfunction and malignant arrythmia.^46^ Persistent symptoms may include hypotension, tachycardia, palpitations, dyspnoea, and arrhythmias,^47^, ^48^, ^49^, ^50^ with some patients requiring hospital readmission.^51^ There are increased risks of incident cardiovascular disease, such as cerebrovascular disorders (stroke/transient ischaemic attacks), dysrhythmia (atrial fibrillation/sinus tachycardia), ischaemic heart disease (acute coronary disease/myocardial infarction/angina), thromboembolic disease (pulmonary embolism/deep vein thrombosis), heart failure, pericarditis, myocarditis, cardiac arrest, and cardiogenic shock.^49^^,^^52^, ^53^, ^54^, ^55^, ^56^ Long-term sequelae may include increased cardiometabolic demand, myocardial fibrosis or scarring, arrhythmias, tachycardia, and autonomic dysfunction, as in POTS.^6^ Increased cardiometabolic demand may be associated with reduced cardiac reserve, corticosteroid use, and dysregulation of the renin–angiotensin–aldosterone system.

Consultation with the cardiology team is vital to ensure that dental treatment is undertaken in a safe manner, considering the risk of cardiac emergency episodes and bacteraemia.^52^ Initial evaluation with noninvasive technology (such as electrocardiography, echocardiography, laboratory testing for C-reactive protein and troponin) is recommended.^54^ Cardiac emergency episodes that may occur in patients during dental treatment include syncope (see POTS in the *neurologic* complication section), coronary ischaemic syndrome, and cardiac arrest*.*^57^ Precautionary measures to prevent cardiac emergency events include assessment and monitoring of vital signs during procedures, as recommended by Chakraborty et al,^7^ as well as the delivery of adequate pain control and stress-reduction measures.^57^

### Effect of COVID-19 on the haematologic system and how it impacts dental management

Thromboembolic events that include segmental pulmonary embolism, intracardiac thrombus, thrombosed arteriovenous fistula, and ischaemic stroke have been noted in less than 5% of COVID-19 survivors.^58^ This COVID-19–associated coagulopathy is consistent with patients’ hyperinflammatory and hypercoagulable state.^6^ The hyperinflammatory response induces endothelitis,^59^ but it is not clear how long endotheliitis can persist in the convalescent phase. Laboratory tests confirmed that patients with elevated D‐dimer (≥0.5 mg/L) and presenting with cardiac injury (see *cardiovascular complication* section) are more prone to coagulation disorders.^60^ Because of the risk of venous thromboembolism in hospitalised COVID‐19 patients lasts up to 90 days following discharge, they receive pharmacologic thromboprophylaxis with low-molecular-weight heparin (LMWH) over unfractionated heparin, unless the risk of bleeding outweighs the risk of thrombosis.^61^

Direct oral anticoagulants and LMWH are sometimes considered for extended thromboprophylaxis in selected cohorts of “risky” patients. Thus, dentists are not expected to manage the haemostasis effect of this drug in the dental office.^6^ However, the impact of extended thromboprophylaxis on dental surgical procedures shall be borne in mind with their management similar to that described under the *neurologic complication* section.

Regular monitoring of blood results and evaluation of the individualised thrombotic risk based on comorbidities and coagulation profile are essential for both postacute and long COVID to provide a tailored therapeutic application.^62^ Invasive dental procedures should be deferred if the patient is deemed to be at high risk.

### Effect of COVID-19 on the renal system and how it impacts dental management

Kidney involvement is not uncommon in COVID-19. Renal complications encountered include proteinuria, haematuria, electrolyte disturbances, reduced glomerular filtration rate (GFR) and, more significantly, acute kidney injury (AKI). AKI is associated with a higher mortality rate, with intensive care unit admission and the need for mechanical ventilation.^63^^,^^64^ The incidence of AKI is variable, ranging between 4.2% and 71.2%.^65^, ^66^, ^67^, ^68^, ^69^, ^70^ Two recent systematic reviews reported a pooled incidence rate of 6% and 11%, respectively, with subgroup analysis indicating that the US population experienced more cases than the Chinese population.^71^^,^^72^

Dentists treating patients recovered from COVID-19 need to be aware that there may be residual renal dysfunction; serum creatinine remained elevated for 47% of COVID-19 patients upon hospital discharge.^67^ In a multicentre observational study, partial recovery from renal deterioration was observed in 17.2% of patients, and this finding was more common amongst patients with preexisting chronic kidney disease.^66^ Postacute sequelae were further corroborated by the observation that COVID-19 survivors had greater longitudinal estimated GFR reduction than noninfected controls.^64^

Compromised renal function can predispose to infection; hence, any orofacial infection must be managed promptly by the removal of the infection source, supplemented with culture and sensitivity testing and appropriate antibiotic prescription as needed. There was no evidence, however, advocating the use of a prophylactic antibiotic to prevent infective endocarditis or endarteritis involving vascular access in patients undergoing hemodialysis.^73^

For patients receiving renal replacement therapy, a few issues must be taken into consideration. Due to concomitant use of antiplatelet/anticoagulant medications and, to a lesser extent, uraemia-induced bleeding disorder, there is a greater risk of bleeding tendencies during and following invasive dental procedures. The dentist should perform coagulation screening prior to dental extractions. Antiplatelet/anticoagulant should not be altered without explicit instructions by the patient's physician or nephrologist. Any elective treatment should be scheduled on a nondialysis day. Local haemostatic agents should be made available and used during invasive dental procedures.

As mentioned earlier, AKI superimposed on COVID-19 survivors with concomitant chronic kidney disease entailed greater risk of residual renal impairment.^66^ When renal function tests show severe impairment (GFR< 50 mL/min), elective dental care should be postponed. Any urgent dental treatment should be carried out in a hospital setting after consultation with the patient's nephrologist and with constant monitoring of patient's creatinine and urine output. When GFR decreases to below 50 mL/min, drug toxicity becomes a major concern. In such cases, either drug dosage is reduced or the interval between administrations is prolonged.^74^ Nephrotoxic drugs such as aspirin, NSAIDs, acyclovir, aminoglycosides, amphotericin, sedatives, muscle relaxants, and tetracycline should be avoided.^75^ When opioids such as morphine, codeine, tramadol, meperidine, and propoxyphene are used, either reduction in dosage or increase in dose interval are required. Acetaminophen (paracetamol) is generally safe due to primary hepatic metabolism. However, the kidneys contribute to glucuronidation of acetaminophen as well.^76^ If GFR falls below 10 mL/min, the dosing interval of acetaminophen should be increased to every 8 hours. Antibiotics that are safer for this group of patients are penicillin and its derivatives, clindamycin, and cephalosporins. Long-term NSAID use at high doses could lead to renal damage and hypertension.^77^ Some NSAIDs that can be considered for patients with renal disease are sulindac, nabumetone, and etodolac because their effects on renal prostaglandins are not as profound.^78^ However, NSAIDs are strictly prohibited amongst predialysis and renal transplant patients.^79^

Azithromycin, erythromycin, clindamycin, doxycycline, and penicillin V are some antibiotics that do not require dose adjustments due to their extensive hepatic metabolism and biliary excretion.^78^ For other antibiotics, either dosage or dose interval adjustments are needed once GFR decreases to below 50 mL/min, preferably after consulting with the patient's physicians.

Local anaesthetic agents such as 2% lidocaine and 2% mepivacaine can be safely used if serum creatinine is less than 2 mg/dL. If possible, the dentists should limit the use of local anaesthesia to 2 carpules, which should be sufficient to achieve adequate anaesthesia for most dental procedures.^75^ The recommended dose adjustment for some commonly prescribed drugs in dentistry according to the patient's GFR is summarised in Table 4.^78^^,^^79^

**Table 4:** Dose adjustment of commonly prescribed drugs in dentistry based on glomerular filtration rate.^78^^,^^79^

Drug	Dose adjustment		
	Normal GFR	GFR between 10 and 50 mL/min	GFR <10 mL/min
**ANTIBIOTICS**			
Amoxicillin	250–500 mg/8 h	Every 8–12 h	Every 24 h
Amoxicillin/clavulanate	250–500/8 h, 875 mg/12 h	Every 8–12 h (do not use 875-mg formulation if GFR < 30 mL/min)	Every 24 h
Clindamycin	300 mg/8 h	No dose adjustment needed	No dose adjustment needed
Cephalexin	250–500 mg/6 h	Every 12 h	Every 24 h
Tetracycline	Best to avoid		
Doxycycline	100 mg/12 h, 20 mg/24 h (host modulation)	No dose adjustment needed	No dose adjustment needed
Erythromycin	250–500 mg/6 h	No dose adjustment needed	No dose adjustment needed
Metronidazole	250–500 mg/8 h	Every 8–12 h	Every 12–14 h
Azithromycin	500 mg/24 h, 3 days	No dose adjustment needed	No dose adjustment needed
**ANALGESICS**			
Acetaminophen	500–1000 mg/6 h	No dose adjustment needed	Every 8 h
Aspirin	Best to avoid		
Ibuprofen	Best to avoid		
Dihydrocodeine	10–30 mg/4–6 h	Decrease normal dose by 25%	Decrease normal dose by 25%
**ANTIFUNGAL**			
Fluconazole	100 mg/24 h	Normal dose	Decrease normal dose by 50%

GFR, glomerular filtration rate.

### Effect of COVID-19 on the gastrointestinal system and impact on dental management

Studies reported that up to 70.3% of patients demonstrated faecal viral RNA shedding even after negative respiratory specimens, with higher numbers in the paediatric population.^80^^,^^81^ Thus, the presence of diarrhoea in an otherwise asymptomatic person would warrant further investigation and raise a high index of suspicion.^82^ Such an inquiry shall be added to the medical history-taking of a dental patient.^7^

SARS-CoV-2 infection can lead to liver injury, with facial skin darkening and increased pigmentation in some patients who recovered from severe COVID-19.^83^ The causes were ascribed to liver dysfunction causing increased melanin production, adrenocortical hypofunction, or an increase in iron levels leading to a darkened face. COVID-19 patients with a darkened face may still continue viral shedding, and their dental case should be treated as non-urgent and elective and flagged.

Last, COVID-19 patients with digestive symptoms more commonly have prolonged prothrombin time (PT) than those with respiratory symptoms. The increased bleeding tendency with prolonged PT indicates the need for local measures for haemostasis following invasive dental procedures, as described earlier in the *neurologic* section. Last, hepatotoxic drugs such as acetaminophen should be reduced or avoided when a patient is diagnosed with liver injury. The expert advice of a gastroenterologist must be sought.

### Effect of COVID-19 on the endocrine system and how it impacts dental management

The endocrine system can be affected significantly by COVID-19.^84^ It has been reported that patients with adrenal insufficiency, such as those with Addison's disease and adrenal hyperplasia, are at higher risk for contracting COVID-19.^84^ Similarly, the effect of COVID-19 on the thyroid and the parathyroid glands has been highlighted, but with few documented cases so far.^85^

Diabetes mellitus is considered an important risk factor associated with COVID-19.^86^, ^87^, ^88^, ^89^ In addition, severe obesity with a body mass index (BMI) of over 40 kg/m^2^, even in young patients, has been associated with increased morbidity and mortality.^90^ In some COVID-19 patients, there is increased expression of ACE-2 on the cells of the exocrine as well as islets of pancreas^91^ associated with elevated serum amylase/lipase levels suggestive of pancreatic injury.^92^ Another theory is related to the impairment of the immune system in patients with diabetes. Defective neutrophilic degranulation, phagocytosis, and complement activation have been suggested to be causative factors for viral infection.^90^^,^^93^ Thus, monitoring and maintaining the normal physiologic level of glucose is important.^94^

Dental treatment of patients with diabetes during COVID-19 recovery remains unchanged, with emphasis on good oral hygiene mainenance.^95^ A thorough case history includes a review of the status of the course of medications, in-house glucose monitoring, and frequency of hypo- and hyperglycemic episodes.^96^ Fasting blood glucose values should range between 4.0 and 7.0 mmol/L, and a range of 5.0 to 10.0 mmol/L is considered acceptable postprandially. The patient should be monitored regularly to prevent the occurrences of any oral infection.^96^^,^^97^

A study conducted in Taiwan had suggested that patients with diabetes had a higher risk of experiencing pneumonia, but patients who received intensive periodontal treatment showed otherwise. This study highlighted that it is essential to control both the diabetic state as well as periodontal health to avoid serious complications of COVID-19.^98^

### Other general management

It is generally acknowledged that COVID-19 is asymptomatic or milder in children and adolescents. Data on long COVID in children and adolescents are still scarce, with an estimated incidence of 5% to 10% due to heterogenicity of symptoms reported.^99^^,^^100^ Behnood et al identified 101 symptoms in children and young people.^101^ Their meta-regression showed that in adolescents, underlying comorbidities and female sex were associated with increased risk of persistent symptoms. Loss of smell, headaches, cognitive difficulties, and sore eyes and throat occurred between 2% and 8% more than the uninfected ones. There is no literature available regarding dental management in this group of patients; we recommend that dentists follow the recommendation for adults, with adjustment according to body weight when prescribing medications.

Vaccination (including booster) has been hailed as the way forwards for protecting both children and adults from the consequences of COVID-19. After COVID-19 vaccination, a 2-week period is usually required to mount adequate immunity against SARS-CoV-2. The dental health care professional should be aware of any systemic and/or orofacial adverse effects from vaccination. The presence of side effects such as headache, fever, or myalgia should be factored in when preparing a patient for dental procedures. Non-urgent procedures should be deferred where possible. Facial swelling from allergic reaction and Bell's palsy have been reported,^102^ so these patients shall be referred accordingly. There are insufficient data to indicate whether the type of vaccine used should influence decision-making when planning dental treatment.

There is a segment of population who do not wish to receive vaccination or who are not fit to do so. The infectivity rate from unvaccinated individuals is estimated to be ∼3 times higher compared to that of vaccinated individuals.^103^ It is thus important to put in place measures to protect them from cross-infection. These include optimal infection control practices, hand hygiene, adequate personal protective equipment, social distancing, and staggered schedules.

Breakthrough infection is defined as COVID-19 disease occurring in fully vaccinated individuals. They are generally milder or asymptomatic, although there is still the possibility for transmission. A recent study showed that the rate of breakthrough infections is extremely low (0.73%) and not associated with any comorbidities.^104^ In addition, one recent study reported that people who had been fully vaccinated against COVID-19 were around half as likely to experience long COVID symptoms.^105^ Nevertheless, non-urgent dental procedures must be deferred.

Last, there have been multiple variants being reported, including Omicron. Spike protein mutations can change the binding affinity to the ACE2 receptor for the vaccine-mediated antibody produced as well as for the virus variant. This favours reinfection and decreases the efficacy of vaccines.^106^ In addition, as the world is entering endemic stage, future infection is expected to continue, along with the long COVID issue. Thus, all suggestions described above may be applicable to future COVID-19 patients infected by different variants.

**Fig. 3:**
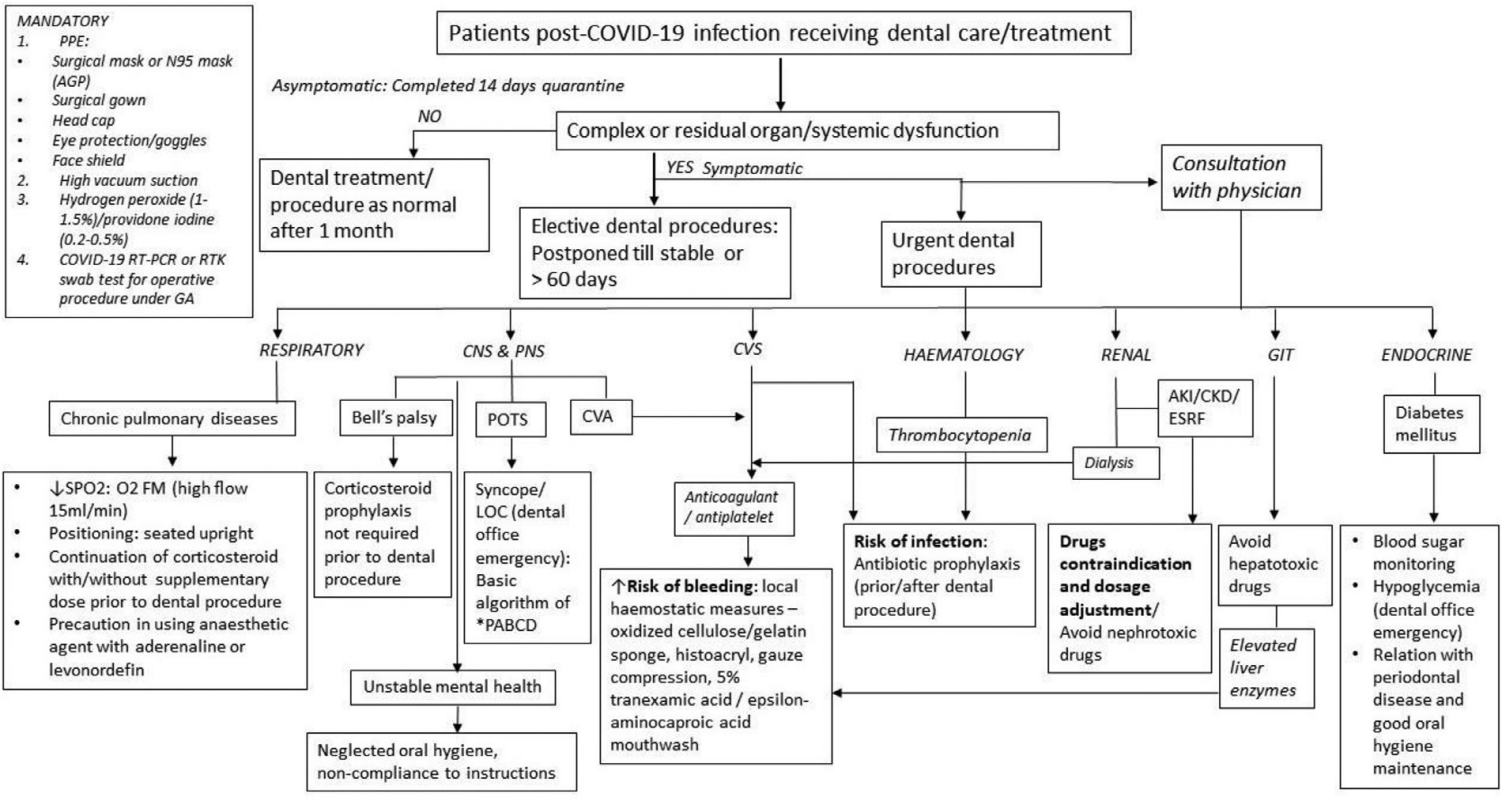
Algorithm summarising the treatment of patients with COVID-19 postrecovery for dental procedures by general dental practitioners. PPE, personal protective equipment; CNS, central nervous system; O2 FM, oxygen face mask; CVA, cerebrovascular accident; CVS, cardiovascular system; GIT, gastrointestinal; POTS, postural orthostatic tachycardia syndrome; PNS, peripheral nervous system; LOC, loss of consciousness; AKI/CKD, acute kidney injury/chronic kidney disease; PABCD, positioning, airway, breathing, circulation, differential diagnosis, drugs and defibrillation. *Note.* It is important to update the patient's medical status during each visit. Basic vital signs, random blood sugar level, and oxygen saturation level need to be obtained. Consultation with physicians denotes the need to consult the respiratory physicians, neurologists, cardiologists, haematologists, urologists, and/or endocrinologists when the need arises. Additional testing such as radiography, electrocardiography, echocardiography, and blood and urine investigation may be needed following consultation.

In summary, this concise review covers the treatment of patients with pulmonary, cardiovascular, haematologic, renal, gastrointestinal, endocrine, and neurologic system complications that result from COVID-19 infection. An algorithm that summarises the management of these patients is provided in Figure 3.

## Author contributions

1.Ngeow WC: Conception, design, drafting, revising all the sections of the article, reviewing, and approving the final version to be published.
2.Tang L: Drafting, revising, referencing formatting, contributing to the “introduction” and “respiratory system” sections of the article, reviewing, and approving the final version to be published.
3.Ho JY: Drafting; revising; contributing to the figure, “introduction,” and “renal system” sections of the article; reviewing; and approving the final version to be published.
4.Tay HW: Drafting, revising, contributing to the figure and “central nervous system” sections of the article, reviewing, and approving the final version to be published.
5.Sekar K: Drafting, revising, contributing to the “haematologic system” section of the article, reviewing, and approving the final version to be published.
6.Ahmad MS: Drafting, revising, contributing the to tables and “cardiovascular system” section of the article, reviewing, and approving the final version to be published.
7.Wong RCW: Drafting, revising, contributing to the “gastrointestinal system” section of the article, reviewing, and approving the final version to be published.
8.Marla V: Drafting, revising, contributing to the “endocrine system” section of the article, reviewing, and approving the final version to be published.
